# Concentration Scales and Solvation Thermodynamics: Some Theoretical and Experimental Observations Regarding Spontaneity and the Partition Ratio

**DOI:** 10.3390/e26090772

**Published:** 2024-09-10

**Authors:** Diego J. Raposo da Silva, Jéssica I. R. de Souza, Ricardo L. Longo

**Affiliations:** 1 Departamento de Química Fundamental, Universidade Federal de Pernambuco, Recife 50740-540, PE, Brazil; jessica.itaiane@ufpe.br; 2 Escola Politécnica de Pernambuco, Universidade de Pernambuco, Campus Benfica, Recife 50702-001, PE, Brazil

**Keywords:** concentration scale, solvation process, experimental NMR, acid–base titration

## Abstract

The solvation thermodynamics (ST) formalism proposed by A. Ben-Naim is a mathematically rigorous and physically grounded theory for describing properties related to solvation. It considers the solvation process as the transfer of a molecule (“solute”) from a fixed position in the ideal gas phase to a fixed position within the solution. Thus, it removes any contribution to the solvation process that is not related to the interactions between this molecule and its environment in the solution. Because ST is based on statistical thermodynamics, the natural variable is number density, which leads to the amount (or “molar”) concentration scale. However, this choice of concentration scale is not unique in classical thermodynamics and the solvation properties can be different for commonly used concentration scales. We proposed and performed experiments with diethylamine in a water/hexadecane heterogeneous mixture to confront the predictions of the ST, based on the amount (or “molar”) concentration scale, and the Fowler–Guggenheim formalism, based on the mole fraction scale. By means of simple acid–base titration and ^1^H NMR measurements, it was established that the predictions of differences in the solvation Gibbs energy and the partition ratio (or “partition coefficient”) of diethylamine between water and hexadecane are consistent with the ST formalism. Additionally, with current literature data, we have shown additional experimental support for the ST. However, due to the arbitrariness of the relative amount of solvents in the partition ratio, the choice of a single concentration scale within the classical thermodynamics is still not possible.

## 1. Introduction

*Solvation thermodynamics* (ST) can be summarized in two aspects: the *decomposition* of the contributions to the chemical potential of a substance; and the *definition* of a process. The chemical potential measures the influence of a substance on the Gibbs energy of a system, which is the state function that determines the stability of open systems. Therefore, the chemical potential of a substance i is related to how relevant it is to drive the system to equilibrium, either physical (e.g., transfer between phases, phase transitions, the transport across membranes of cells) or chemical (e.g., reactions in the bulk or at interfaces). It can also be understood as the (non-expansion) reversible work associated with the transfer of species i from an infinity distance to the interior of a given phase α. Different energy contributions are involved in this transfer process. As shown by A. Ben-Naim, the chemical potential of a substance i, $\mu_{i,\alpha }$, can be partitioned into two terms: one related to the *interactions* and another related to the *liberation* [1,2,3,4,5,6]:

- *Interaction*: the work required to insert a particle (the specific conformer of a molecule, for instance) at a fixed position and orientation within α, so it is unable to move because it lacks the translational degree of freedom (unlike the other particles in α);
- *Liberation*: the energy required to provide translational energy to the inserted molecule to remove the limitation that prevents it from sampling the whole volume of the system, and the energy related to making it indistinguishable from other particles of the same kind, if there are any (because, while the particle was fixed, it was distinguishable).

The interaction term, called the *pseudo-chemical potential*, $\mu_{i,\alpha }^{*}$, is related to the interactions between i and all particles within α. The sampling of the volume of the system by particle i and the remaining contributions of the liberation energy are not influenced by such interactions and should not be considered in the solvation process. Thus, within the solvation thermodynamics (ST) theory, the *Gibbs energy of solvation* (SGE) per molecule is defined as [1,2,3,4,5,6]

(1) $$\Delta G_{i,\alpha }^{*}:=\mu_{i,\alpha }^{*}-\mu_{i,\mathrm{ig}}^{*}.$$

with $\mu_{i,\mathrm{ig}}^{*}$ being the pseudo-chemical potential of species i in the ideal gas phase. Notice that phase α can be a pure solvent or a mixture of arbitrary composition, which gives a generality to this quantity, and no distinction or definition of solute and solvent is necessary. Basically, $\Delta G_{i,\alpha }^{*}$ quantifies the work associated with the transfer of i from the ideal gas (a specific conformer at a fixed position and orientation) to α (also at a fixed position and orientation).

Once defined, one must describe the ways to measure and calculate the solvation energy. Before the ST was developed, solvation energy was estimated through the analysis of the transference of a molecule in ideal gas to a solution under standard conditions, that is, ideal gases and dilute solutions. Having established the solvation equilibrium for these cases, the solute partial pressure and solution concentration (in some scale) were used to determine the *standard solvation Gibbs energy* (SSGE). Giving several possibilities for measuring the amount of substance in gas (e.g., partial pressure, molar concentration) and in solution (mass, volume, number, “molar”, or molal concentrations, mole fraction, etc.), different SSGEs were measured for each choice, and authors from different schools defended the use (and the actual meaning of solvation) of one or another choice. These interpretations are discussed in the Supplementary Materials, but a specific scale shall be presented here as an example. Notice that IUPAC recommends [7] an amount-of-substance concentration, or briefly put, an *amount concentration* or simply a *concentration,* for the previously called “molar concentration” or “molarity”. The concentration is denoted as $c_{B}$ or [B] and defined as $c_{B}=n_{B}/V$ ($mol\;m^{-3}$), where V is the volume of the solution and $n_{B}$ is the amount (of substance) of B, which should not be called “number of moles” [7], and is given by $N_{B}/L$, with $N_{B}$ being the number of (entities) B and L (or $N_{A}$) being the Avogadro constant. The molality $m_{B}$ or $b_{B}$ is defined as $b_{B}=n_{B}/m_{A}$ ($mol\;{kg}^{-1}$), where $m_{A}$ is the mass of solvent A. The mole fraction or amount-of-substance fraction or amount fraction is denoted by $x_{B}$ (for condensed phases) or $y_{B}$ (for gaseous mixtures) and defined as $x_{B}=n_{B}/\sum_{i}n_{i}$, with the summation running over all components in the solution. So, the SSGEs of solute i are $\Delta {\bar{G}}_{i,\alpha }^{o}(c-c)$, $\Delta {\bar{G}}_{i,\alpha }^{o}(p-b)$, and $\Delta {\bar{G}}_{i,\alpha }^{o}(p-x)$ in the amount concentration, molality, and amount fraction (or mole fraction) concentration scales, respectively. It can be shown that the SGE $\Delta {\bar{G}}_{i,\alpha }^{*}$ is numerically equivalent to the SSGE $\Delta {\bar{G}}_{i,\alpha }^{o}(c-c)$ [2,3,4] (see Supplementary Materials).

SSGEs can be promptly generalized to the transfer of species i from phase α to β as $\Delta_{\alpha }^{\beta }{\bar{G}}_{i}^{o}\left(u-u\right)$, where u denotes the concentration scale. As a result, the transference energy of i between two immiscible solvents 1 and 2 is given by $\Delta_{1}^{2}{\bar{G}}_{i}^{*}$ and $\Delta_{1}^{2}{\bar{G}}_{i}^{o}\left(c-c\right)$ within the ST formalism and in the amount concentration scale, which are numerically equal, and by $\Delta_{1}^{2}{\bar{G}}_{i}^{o}\left(x-x\right)$ in the amount fraction concentration scale, for instance. By using the transformations between standard states, it can be shown that [2,3,4] (see Supplementary Materials):

(2) $$\Delta_{1}^{2}{\bar{G}}_{i}^{*}=\Delta_{1}^{2}{\bar{G}}_{i}^{o}\left(c-c\right)=\Delta_{1}^{2}{\bar{G}}_{i}^{o}\left(x-x\right)+RT\ln\left({\bar{V}}_{2}/{\bar{V}}_{1}\right).$$

where ${\bar{V}}_{\alpha }$ is the molar volume of phase α (1 or 2). The term in Equation (2) involving the ratio of the molar volumes does not depend on the relative interactions between i and the particles in either phase. However, it does create a crucial difference between the values of the solvation energies based on the two approaches, particularly when ${\bar{V}}_{2}\gg {\bar{V}}_{1}$ or ${\bar{V}}_{2}\ll {\bar{V}}_{1}$. This difference is also present when other concentration scales are chosen, such as molality. The first equality in Equation (2) represents the equivalence of the value of $\Delta_{1}^{2}{\bar{G}}_{i}^{o}$ in the amount (or “molar”) concentration to the ST value, which becomes the most adequate or “natural” concentration scale for describing the solvation and partition within the ST formalism. Because ST is still unknown to many scientists and might not be universally accepted, and since the choice of the concentration scale within classical thermodynamics is arbitrary, the issue of which concentration scale is the most adequate for describing solvation and partition is still being debated. Approaches to settle this debate have been proposed, such as in the paper of Auton and Bolen [8]:

*To rigorously test the question of preference regarding molal- and molar-based transfer free energies or whether neither is adequate in all cases, experiments performed in solvents of widely differing densities will be required.*

In a paper where Moeser and Horinek [9] delve into the different concentration scales, they reach the following conclusion:

*As explained in the paper at hand, statistical thermodynamics answers this question without the need for further experiments: the molarity scale STFE* [standard transfer free energy] *is the TFE* [transfer free energy] *that quantifies the preference of a solute for one solvent over another.*

The distinction between the concentration scales describing the solvation process becomes even more pronounced when not only the values of the transference energies are different, but also have opposite signs. This means that the solvation preference shifts from one phase to another depending on the choice of the concentration scale.

In this context, we attempted to explore the experimental data available in the literature as well as to provide new experimental data based on two distinct experimental methods to provide some support for the choice of ST and amount concentration scale. Some theoretical support has also been given for this choice. More specifically, we tried to show the following:

(I) Opposite sign predictions between processes based on different concentration scales can be found in several examples, rather than just a few. They are especially prone to misinterpretations regarding the spontaneity of the solvation process, so we show how they can be systematically found, and that the conversion between the predictions (based on the relation between concentrations in ideal solutions) works well, with the theory fitting the experimental comparison between two different sources of solvation energies;
(II) Partition ratios (or “partition coefficients”) can also be used to predict solvation preferences, but also present sign inversions for some substances, hence demanding a consistent choice regarding which scale is used;
(III) Measurements of the partition ratio of diethylamine in water/hexadecane mixtures were performed with two distinct methods (acid–base titration and NMR). The experiments confirmed that the use of mole fraction or amount concentration scales leads to widely different predictions;
(IV) We also discuss some other aspects regarding the merits of the amount concentration scale, such as the present definition of the partition ratio (or “partition coefficient”) by IUPAC and how this scale is advantageous in the study of electrolyte solutions.

## 2. Theoretical Background and Analyses of the Literature Data for Transference

For diluted solutions, concentration scales are readily related to each other and depend on the properties of the solvent s and physical constants. It can be shown that the energy in the mole fraction scale ${∆}_{\mathrm{ig}}^{s}{\bar{G}}_{i}^{o}\left(p-x\right)$ is related to the concentration ${∆}_{\mathrm{ig}}^{s}{\bar{G}}_{i}^{o}\left(p-c\right)$ or ${∆}_{\mathrm{ig}}^{s}{\bar{G}}_{i}^{o}\left(c-c\right)$ and to the molality ${∆}_{\mathrm{ig}}^{s}{\bar{G}}_{i}^{o}\left(p-b\right)$ scales as (see Supplementary Materials):

(3) $$\begin{array}{ll} {∆}_{\mathrm{ig}}^{s}{\bar{G}}_{i}^{o}\left(p-x\right) & ={∆}_{\mathrm{ig}}^{s}{\bar{G}}_{i}^{o}\left(p-c\right)-RT\ln\left(\frac{c^{o}M_{s}}{x^{o}d_{s}}\right)\\ & ={∆}_{\mathrm{ig}}^{s}{\bar{G}}_{i}^{o}\left(p-b\right)-RT\ln\left(b^{o}M_{s}/x^{o}\right)\\ & ={∆}_{\mathrm{ig}}^{s}{\bar{G}}_{i}^{o}\left(c-c\right)-RT\ln\left(\frac{M_{s}p^{o}}{x^{o}RTd_{s}}\right)\\ & =∆{\bar{G}}_{i,s}^{*}-RT\ln\left(\frac{M_{s}p^{o}}{x^{o}RTd_{s}}\right), \end{array}$$

where the solvation energy according to the u and u’ scales, from ideal gas ig to a solute s, respectively, is indicated by ${∆}_{\mathrm{ig}}^{s}{\bar{G}}_{i}^{o}\left(u-u’\right)$. The properties of the solvent are the density, $d_{s}$, and molar mass, $M_{s}$, while $x^{o}=1$, $b^{o}=1\;\mathrm{mol}\;{\mathrm{kg}}^{-1}$, and $c^{o}=1\;\mathrm{mol}\;{\mathrm{dm}}^{-3}$ are the standard mole fraction, molal, and amount concentrations, respectively. The SSGE based on the amount concentration scale for both phases, ${∆}_{\mathrm{ig}}^{s}{\bar{G}}_{i}^{o}\left(c-c\right)$, is numerically equivalent to the SGE defined by the ST formalism $∆{\bar{G}}_{i,s}^{*}$, leading to the last equality in Equation (3) [1,2,3,4,5,6] (see Supplementary Materials). Although these equations provide the relation between any formalism, we will focus on process-*x*, which corresponds to the energy ${∆}_{\mathrm{ig}}^{s}{\bar{G}}_{i}^{o}\left(p-x\right)$, and process-*c*, which is related to ${∆}_{\mathrm{ig}}^{s}{\bar{G}}_{i}^{o}\left(c-c\right)$, because it is equivalent to the predicted energy from ST.

The SSGE according to process-*x* is analogous to the formalism presented by Fowler and Guggenheim [10], as pointed out by Sordo [11]. It is related to process-*c*, and to the ST in ideal dilute solutions, as

(4) $$∆{\bar{G}}_{i,s}^{*}={∆}_{\mathrm{ig}}^{s}{\bar{G}}_{i}^{o}\left(p-x\right)+RT\ln\left(\frac{M_{s}p^{o}}{x^{o}RTd_{s}}\right)={∆}_{\mathrm{ig}}^{s}{\bar{G}}_{i}^{o}\left(p-x\right)+RT\ln\left({\bar{V}}_{s}/{\bar{V}}_{\mathrm{ig}}\right),$$

where ${\bar{V}}_{\mathrm{ig}}=RT/p^{o}$ is the molar volume of an ideal gas at standard pressure $p^{o}$, ${\bar{V}}_{s}=M_{s}/d_{s}$ is the molar volume of the solvent, and $x^{o}=1$. If $p^{o}=1\;\mathrm{atm}=1.01325\times 10^{5}\;\mathrm{Pa}$, adopted herein because it is closely related to the IUPAC recommended value of $10^{5}\;\mathrm{Pa}$, often used in older reports, and if T=298.15 K, then the value of ${\bar{V}}_{\mathrm{ig}}$ is $24.4655\;L\;{\mathrm{mol}}^{-1}$. For water, ${\bar{V}}_{w}=1.807\times 10^{-2}\;L{\;\mathrm{mol}}^{-1}$ at 25 °C [7], and the numerical relation between the SSGEs according to process-*x* and process-*c* is

(5) $$∆{\bar{G}}_{i,s}^{*}={∆}_{\mathrm{ig}}^{s}{\bar{G}}_{i}^{o}\left(p-x\right)-4.272\;\mathrm{kcal}{\;\mathrm{mol}}^{-1},$$

Plenty of data for $∆{\bar{G}}_{i,w}^{*}={∆}_{\mathrm{ig}}^{w}{\bar{G}}_{i}^{o}\left(c-c\right)$ [4] and ${∆}_{\mathrm{ig}}^{w}{\bar{G}}_{i}^{o}\left(p-x\right)$ [12,13] are available, so the validity of Equation (5) can be ascertained, which has indeed been corroborated for all substances in Table 1 within the experimental error. The average value of the difference between ${∆}_{\mathrm{ig}}^{w}{\bar{G}}_{i}^{o}\left(p-x\right)$ and $∆{\bar{G}}_{i,w}^{*}$ is $4.272\;\mathrm{kcal}{\;\mathrm{mol}}^{-1}$, which is in remarkable agreement with Equation (5).

Another relevant aspect regarding Equation (6) is that, for some substances, the values of ${∆}_{\mathrm{ig}}^{s}{\bar{G}}_{i}^{o}\left(p-x\right)$ and ${∆}_{\mathrm{ig}}^{s}{\bar{G}}_{i}^{o}\left(c-c\right)$ have *opposite signs*, which potentially pose *qualitative* discrepancies for describing the spontaneity of the solvation process. Some of the substances that could display such opposite signs are listed in Table 2. For instance, dimethyl ether (DME) presents a value for ${∆}_{\mathrm{ig}}^{w}{\bar{G}}_{i}^{o}\left(p-x\right)$ of $2.38\;\mathrm{kcal}\;{\mathrm{mol}}^{-1}$ (Table 2), while ${∆}_{\mathrm{ig}}^{w}{\bar{G}}_{i}^{o}\left(c-c\right)$ is $-1.89\;\mathrm{kcal}\;{\mathrm{mol}}^{-1}$, which provide the opposite experimental behavior and, most likely, the opposite conclusions regarding the spontaneity of the solvation process.

Notice that the values of the solvation energies require measurements in the gas phase, so their comparisons can be limited. So, an alternative consists of measuring the solubility in different solvents. Indeed, such measurements led to interesting results regarding the solubility of propane in D_2_O and H_2_O [2,5,6]. At 25 °C, the solubility ratio in these two solvents is 1.032 when expressed in mole fraction, suggesting that propane is more soluble in D_2_O than in H_2_O, and it is 0.921 in molality, which is the opposite trend [5]. Using these results, the calculated $\Delta_{H_{2}O}^{D_{2}O}{\bar{G}}_{i}^{o}$ in the mole fraction scale has an opposite sign to that in the molality scale [5]. These contrasting results are due to the choice of measuring the solubility as the amount of solute in a given amount (mole), a given mass, or a given volume of the solvent. So, standard classical thermodynamics cannot provide an answer to in the question of which solvent (D_2_O or H_2_O) has a larger Gibbs energy of solvation. However, by measuring the energy of solvation within ST formalism, this contraction disappears because its value is calculated in the amount (or “molarity”) concentration scale [5].

Measuring the amount of solute in the gas phase or in a solvent can be taxing. An experimentally simpler approach to ascertain the solvation properties of solvents consists of determining the amount of a substance in two immiscible solvents at equilibrium, namely by means of the partition ratio (formerly “partition coefficient”). Thus, in the following section, the experimental results for the partition of diethylamine in the water/hexadecane mixture shall be presented and discussed.

## 3. Experimental Partition of Diethylamine in Water/Hexadecane Mixture

Amongst the substances listed in Table 2, and for all examples in Abraham et al. [14], diethylamine is the one with the highest disparity for the Gibbs energies of transfer. So, it is expected that the values obtained from process-*x* (Fowler–Guggenheim formalism) or process-*c* (solvation thermodynamics) will be very different, way beyond the uncertainty of usual experimental techniques.

The experiments consist in mixing a given amount of diethylamine in a heterogeneous mixture of water and hexadecane with equal volumes, before letting it equilibrate (see Section 6). The amount of solute in the water was determined by acid–base titration and by ^1^H NMR spectroscopy (before and after equilibration). The amount of diethylamine in hexadecane was obtained by the conservation of mass.

The volume of the titrant (potassium biphthalate 0.100 $mol\;L^{-1}$) in three acid–base titrations of the aqueous solution of diethylamine were 8.5, 7.7, and 8.0 mL. So, the initial amount of diethylamine (4.05 mmol) is partitioned, on average, into 3.20 mmol in 20 mL of water and 0.85 mmol in 20 mL of hexadecane. From these values, the usual “partition coefficient” could be determined. However, IUPAC considers the “partition coefficient” to be obsolete [15] and suggest the partition ratio, $K_{D}$, to be defined as “the ratio of the concentration of a substance in a single definite form in the extract to its concentration in the same form in the other phase at equilibrium” [16]. For a water/hexadecane (w/h) system, the partition ratio of diethylamine (d) becomes

(6) $$K_{D}=\frac{c_{d,h}}{c_{d,w}}=\frac{n_{d,h}/V_{h}}{n_{d,w}/V_{w}}≅\frac{n_{d,h}}{n_{d,w}},$$

where $c_{d,h}$ and $c_{d,w}$ are the amount concentrations of diethylamine, at equilibrium, in hexadecane and water, respectively; $n_{d,h}$ and $n_{d,w}$ are the corresponding amounts of diethylamine in hexadecane and water, respectively; and $V_{h}$ and $V_{w}$ are the volumes of the organic and aqueous solutions, respectively. The last equality considers equal volumes of the solvents and because the volume of the solute is much smaller than the solvents (e.g., 0.4 mL versus 20 mL), the volume of the solutions can be approximated by the volume of the solvents. Promptly, we obtained an average value of 0.26 ± 0.15 for the partition ratio of diethylamine in the water/hexadecane system.

By analogy, the following a partition, $K_{X}$, could be proposed when measuring the mole fraction of the solute in the immiscible solvent, namely:

(7) $$K_{X}=\frac{x_{d,h}}{x_{d,w}},$$

where $x_{d,h}$ and $x_{d,w}$ are the amount (or mole) fractions of diethylamine, at equilibrium, in hexadecane and water, respectively. From the measured amounts of diethylamine in hexadecane and water, and from the molar masses (18.01528 and 226.4412 g/mol) and densities (0.99704 and 0.7701 g/mL at 25 °C) of water and hexadecane, an average of 4.2 ± 2.5 for the “mole fraction partition ratio” is obtained.

**Figure 1:**
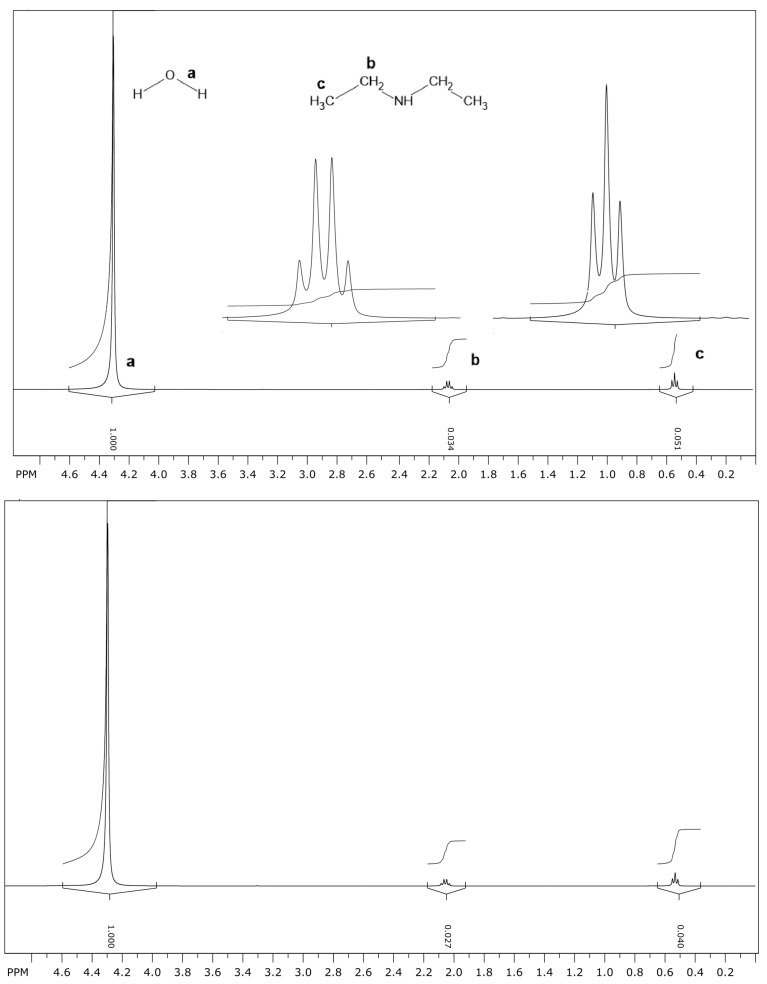
^1^H NMR spectra of diethylamine in water before (**top panel**) and after (**bottom panel**) being mixed with hexadecane at 25 °C. In the top panel, the selected peaks for determining the areas and the assignments are emphasized. The same is applied to the bottom panel.

The amount of diethylamine in the water/hexadecane system has also been determined by ^1^H NMR. This determination is made by comparing the areas of a given peak in the spectra before and after the partition of diethylamine. Examples of these spectra are illustrated in Figure 1, with the assignments and emphasis on the selected peaks for determining the areas.

From the areas in the ^1^H NMR spectra, the amounts of diethylamine partitioned in the water/hexadecane system were determined and yielded a partition ratio $K_{D}$ average value of 0.265 ± 0.047 for both CH_2_ and CH_3_ signals and three experiments. An average value of 4.32 ± 0.76 was determined for the “mole fraction partition ratio” $K_{X}$. These values agree quite well with those obtained from the acid–base titration.

From the value of the partition ratio $K_{D}$, the Gibbs energy of transference with the ST formalism (process-*c*) can be determined as $\Delta_{w}^{h}{\bar{G}}_{d}^{*}=\Delta_{w}^{h}{\bar{G}}_{d}^{o}\left(c-c\right)=-RT\ln K_{D}$, which yields 0.80 ± 0.36 and 0.770 ± 0.040 kcal/mol for acid–base titration and ^1^H NMR, respectively. Similarly, the SSGE for the traditional Fowler–Guggenheim approach (process-*x*) can be calculated from the values of $K_{X}$, yielding −0.82 ± 0.36 (acid–base titration) and −0.847 ± 0.040 (NMR) kcal/mol. These values for the Gibbs energy clearly show the opposite trend and the opposite behavior regarding the spontaneity and preferential solvation. These results are qualitatively and quantitatively consistent with the data in Table 2 and with Equation (2), and were verified to be reliable (compilation in Table 1) for diethylamine in the water/hexadecane system.

## 4. Concentration Scales and Preferential Solvation

Concerning the investigation into solvation, there are instances where the distinction between solute and solvent is difficult or even impossible. Therefore, for a general theory of solvation, it would be desirable to employ a concentration scale that does not require this distinction. On these grounds, the molality scale should be avoided because it makes a direct reference to the mass of the solvent in its definition. The mole fraction scale does not require this distinction in its definition; however, it suffers from ambiguity when used for solutions with at least one component that can be (partially) dissociated, e.g., electrolytes [17] (pp. 31–39). This ambiguity is related to use of the “total number of solute particles” or the “formula weights of solute” [17] (pp. 31–39). To solve this ambiguity, IUPAC defines the amount (or mole) fraction as the amount of a constituent divided by the total amount of all constituents in the mixture, which is equal to the number fraction defined as the number of entities of one constituent divided by the total number of entities in the mixture [18]. Despite its unambiguous definition, the mole fraction scale still suffers from difficulties for a multicomponent mixture with multiple dissociable components, because the dissociation degrees of each dissociable species must be known to calculate the denominator of the mole fraction. On the other hand, the amount (or “molar”) concentration (or “molarity”) scale does not make any reference to solute or solvent, and because it employs the volume of the solution in the denominator for its definition, only the degree of dissociation of the species of interest is needed.

For describing, quantifying, and interpreting the influence of solute–solvent interactions on the activity coefficients, it is desirable to have quantities expressing the concentration that are independent of the solute being treated as solvated or not. Otherwise, the properties derived from these quantities (e.g., chemical potential) will depend on the number of molecules that is solvating the solute [17] (pp. 238–252) and would artificially distinguish between solvent molecules that are solvating the solute and those that are not. Indeed, this issue of the dependence on the solvation number of the solute was recognized several decades ago [17] (pp. 238–252) and it arises from the choice of the concentration scale. For mole fraction and molality scales, the quantities expressing the composition will be different if the solute is considered as a solvated species and will also differ on how many molecules are solvating it [17] (pp. 238–252). On the other hand, for the amount or number concentration scale, the quantity describing the composition of the solution, and hence the properties derived from this quantity, is the same regardless of whether the solute is treated as solvated or not. This has been recognized as “an advantage of using the concentration scale”, but it was disregarded because it “is outweighed by the disadvantage that the amount concentration of a solution changes with temperature” [17] (pp. 238–252). We strongly disagree with this assessment because it is indeed a good practice to employ a concentration scale that depends on temperature, since controlling and reporting the temperature would always be required when measuring thermochemical properties. It is noteworthy that, within the ST formalism, these issues surrounding the solvation number of the solute and the distinction of the solvent molecules do not arise for activity coefficients or other solvation-related quantities [19].

Despite these profound distinctions between the concentration scales for describing the solvation process, within standard classical thermodynamics, it is not possible to select one specific scale. However, because IUPAC considers the “partition coefficient” as obsolete and recommends the partition ratio $K_{D}$ based on the amount concentration of the solute into each heterogeneous phase, it appears that the amount (or “molar”) concentration scale has been chosen. Notice that IUPAC does not mention any other quantity to describe this partition, except the partition constant, $K_{D}^{o}$, defined as the ratio of the activity of the solute within each phase at equilibrium [20]. Despite $K_{D}^{o}$ being dependent on the choice of standard states, if the pure solvent and infinite dilution are taken as standard states, then $K_{D}\to K_{D}^{o}$ as the total concentration of dissolved materials decreases [16]. These were the main reasons for choosing the same volume for the solvents when performing the experiments wherein diethylamine was partitioned into the water/hexadecane heterogeneous phases.

**Figure 2:**
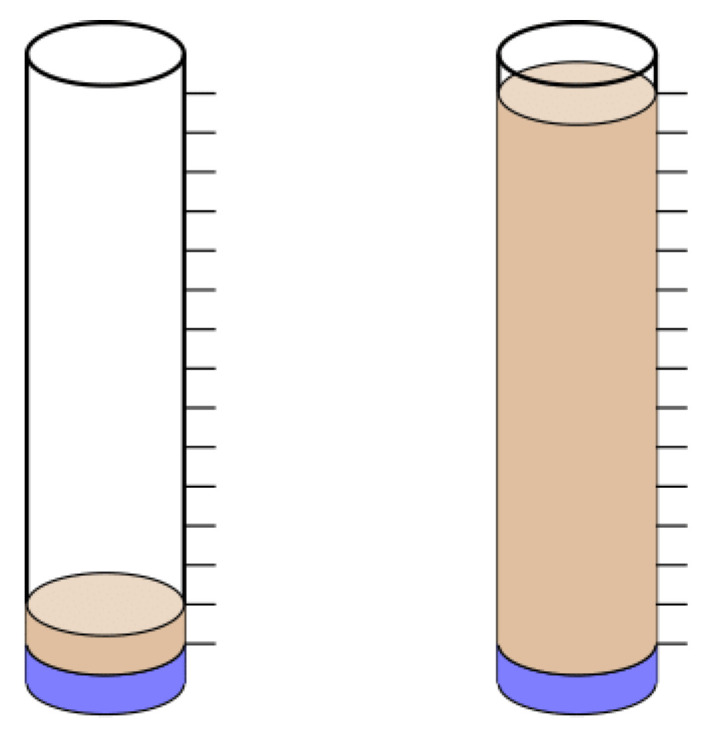
Schematic representation of two possible experiments regarding the diethylamine/hexadecane/water system. On the left panel, 20 mL of water (1.1 mol) is mixed with 20 mL of hexadecane (0.068 mol), and on the right panel, 20 mL of water (1.1 mol) is mixed with 320 mL of hexadecane (1.1 mol). In both cases, the amount of diethylamine (4 mmol) is much smaller than the amount of either solvent.

The choice of volumes for the solvents for measuring the partition of a solute instead of the number of molecules of each solvent also serves to avoid the highly visually asymmetric heterogeneity (ca. 16 times) depicted in Figure 2. This figure approximately shows the volumes of each phase required to provide similar results to equal volumes when considering the partition into phases with the same number of molecules for the water/hexadecane system, which involves a ratio of molar volume of ca. 16. In addition, by choosing to measure the partition into phases with a similar number of molecules, the experimental procedure would require the properties of each solvent (e.g., density, molar mass) to set up the partition and the measurements.

## 5. Conclusions

The experimental results for the partition of diethylamine between the water and hexadecane phases successfully demonstrated the qualitative and quantitative discrepancies between the partition equilibrium and Gibbs energy of transference when employing the amount (“molar”) concentration or the traditional approach (Fowler–Guggenheim) based on mole fraction scale. The results were obtained with distinct methods, namely acid–base titration and ^1^H NMR, which are consistent and quantitatively equivalent within the experimental uncertainties of each technique. The choice of considering the volumes of each phase to measure the partition was based on the definition of the partition ratio by IUPAC to the detriment of the partition coefficient, which does not specify the choice of volume, mass, or number of molecules of each solvent. So, by only defining the partition ratio, which is based on the amount concentration, for quantifying the partition of a substance into heterogeneous phases, IUPAC appears to have made a choice to describe the solvation process by means of the (amount) concentration scale. This is an interesting choice because it is fully compatible with the solvation thermodynamics, a statistical mechanics-based formalism. As in the case of choosing the Boltzmann constant as one of the fundamental defined constants in the most recent International System of Units, instead of the (molar) gas constant, we wonder whether the choice of partition ratio by IUPAC was also motivated by a microscopic description of macroscopic systems.

## 6. Experimental Procedure

### 6.1. Acid–Base Titration

In a 100 mL separating funnel, 20 mL of pure water, containing small amounts of K_2_CO_3_ to adjust the pH to 7.0, was mixed with 20 mL of hexadecane. Then, 420 μL of diethylamine (4.05 mmol) was added to the mixture and after agitation, it was kept for 3 days at room temperature (25–28 °C) for phase transfer equilibration. Afterwards, 5 mL of the aqueous phase was transferred to an Erlenmeyer, followed by the addition of 15 mL of distilled water and a few drops of phenolphthalein, and the volumetric analysis was carried out with potassium biphthalate (KHP) 0.100 mol/L aqueous solution using 1% phenolphtalein ethanolic solution (ca. 1 mL) as an indicator [12,21]. This procedure was repeated 3 times and yielded 8.5, 7.7, and 8.0 mL, with an average of 8.1 ± 1.0 mL (95% confidence interval based on Student distribution) as the volume of the titrant, which corresponds to 3.4, 3.08, and 3.2 mmol (average 3.2 ± 0.4 mmol) of diethylamine in 20 mL of water.

### 6.2. ^1^H NMR Experiments

All NMR experiments were performed on an Agilent 400 MHz spectrometer, operating at 25 °C, with resonance frequencies of 399.75 MHz for ^1^H, without a water signal suppression scheme. The experiments were carried out in an NMR capillary tube, and recorded using the s2pul sequence with broadband Waltz-16 ^1^H decoupling, a total of 32k complex data points, and an acquisition time of 1.52 s.

The procedure consists of mixing 20 mL of distilled water (pH 7) with 420 μL of diethylamine, and then, 350 μL of the solution was transferred to the NMR tube and the ^1^H NMR spectrum was acquired. The tube was removed from the equipment and 350 μL of hexadecane was added, followed by vigorous mixing for 1 min, and an equilibrium time of 1 h at 25 °C. The tube was inserted into the equipment and the ^1^H NMR spectrum in water was acquired. This procedure was repeated 3 times. Notice that there is a small amount of hexadecane in the water, which was confirmed by measuring the pure hexadecane spectrum beforehand, but not enough to compromise the analysis, because there is only one signal for each proton in the water, not two. The areas of the signals of the CH_2_ and CH_3_ protons before and after the partition were used to determine the amount of diethylamine in the water and, by mass conservation, in hexadecane. This determination can be achieved by considering that the area of each signal is proportional to the amount of diethylamine. Because the volume of water and other parameters are the same, the amount of diethylamine in the water can be calculated as $n_{d,w}=n_{d,w}^{0}(A_{d,w}/A_{d,w}^{0})$, where $n_{d,w}^{0}$ is the amount before the partition, and $A_{d,w}^{0}$ and $A_{d,w}$ are the areas of a given signal (CH_2_ or CH_3_) before and after the partition, respectively. As a result, the partition ratio becomes $K_{D}=\left(A_{d,w}^{0}-A_{d,w}\right)/A_{d,w}$ for the same volume of solvents. The areas $A_{d,w}^{0}$ and $A_{d,w}$ for the signals of the CH_2_ and CH_3_ protons in three experiments are presented in Table 3.

## Figures and Tables

**Table 1:** Values of $∆{\bar{G}}_{i,w}^{*}$ (Ben-Naim [3]) and ${∆}_{\mathrm{ig}}^{w}{\bar{G}}_{i}^{o}\left(p-x\right)$ (Abraham et al. [13]) energies for different substances in water (w) and their differences. All values are in $\mathrm{kcal}{\;\mathrm{mol}}^{-1}$.

Solute (i)	$∆{\bar{G}}_{i,w}^{*}$	${∆}_{\mathrm{ig}}^{w}{\bar{G}}_{i}^{o}\left(p-x\right)$	$∆∆{\bar{G}}_{i}$ ^1^
Methane	2.005	6.27	4.265
Ethane	1.833	6.09	4.257
Propane	1.959	6.23	4.271
Butane	2.083	6.34	4.257
2-Methylpropane	2.323	6.59	4.267
Methanol	−5.10	−0.83	4.270
Ethanol	−5.05	−0.73	4.320
Ammonia	−4.305	−0.03	4.275
Methylamine	−4.569	−0.29	4.279
Ethylamine	−4.507	−0.23	4.277
Propylamine	−4.401	−0.12	4.281
Butylamine	−4.302	0.03	4.332
Diethylamine	−4.080	0.19	4.270
Triethylamine	−3.042	1.05	4.092
Benzene	−0.767	3.39	4.157
Toluene	−0.882	3.48	4.362
Ethylbenzene	−0.789	3.61	4.399
Neon	2.671	6.94	4.269
Argon	2.002	6.27	4.268
Krypton	1.658	5.93	4.272
Xenon	1.335	5.60	4.265

^1^ $∆∆{\bar{G}}_{i}={∆}_{\mathrm{ig}}^{w}{\bar{G}}_{i}^{o}\left(p-x\right)-∆{\bar{G}}_{i,w}^{*}$ and the average of the differences is $-4.272\;\mathrm{kcal}{\;\mathrm{mol}}^{-1}$ at 298 K.

**Table 2:** Selected substances with solvation energy (second column) and transfer energy between water and hexadecane (third column) with opposite signs of energies for process-x and process-c. The data, in $\mathrm{kcal}{\;\mathrm{mol}}^{-1}$, were gathered from a compilation by Abraham et al. [14].

Solute (i)	${∆}_{\mathrm{ig}}^{w}{\bar{G}}_{i}^{o}\left(p-x\right)$	${∆}_{w}^{h}{\bar{G}}_{i}^{o}\left(x-x\right)$
Ethyne	4.26	
Propyne	3.79	
Benzene	3.39	
Propanone	0.46	−0.24
Butanone	0.56	−1.06
Cyclopentanone		−1.21
Formaldehyde	1.52	
Acetaldehyde	0.77	
Propanal	0.83	−0.69
Dimethyl ether	2.38	
Diethyl ether	2.68	
Methyl formate	1.49	−0.86
Methyl acetate	1.13	−1.18
Methyl propanoate	1.34	
Butan-1-ol		−0.48
Pentan-1-ol		−1.32
Pentanoic acid		−0.09
Hexanoic acid		−0.77
Propylamine		−0.18
Butylamine	0.03	−0.98
Diethylamine	0.19	−0.84
Pyridine		−1.06
Nitromethane	0.25	−0.21
Nitroethane	0.56	−1.17
Acetonitrile	0.38	
Propanonitrile	0.43	−0.61
Chloromethane	3.72	
Chloroethane	3.64	
Bromomethane	3.46	
Iodomethane	3.37	
Fluoromethane	4.06	
Benzyl chloride	2.35	
Benzyl bromide	1.90	
Fluorobenzene	3.48	
Chlorobenzene	3.13	
Bromobenzene	2.81	
Iodobenzene	2.53	
Methanethiol	2.91	
Ethanethiol	3.13	
Dimethyl sulfide	2.72	
Thiophenol	1.72	

**Table 3:** Areas in the ^1^H NMR spectrum before ($A_{d,w}^{0}$) and after$\;(A_{d,w}$) the partition of diethylamine in the same volume of water and hexadecane.

Experiment	$A_{d,w}^{0}$ (CH_2_)	$A_{d,w}^{0}$ (CH_3_)	$A_{d,w}$ (CH_2_)	$A_{d,w}$ (CH_3_)
1	0.022	0.032	0.017	0.025
2	0.034	0.050	0.027	0.040
3	0.045	0.068	0.036	0.054

## Data Availability

The original data presented in the study are openly available in the Supplementary Materials or upon request from the authors.

## Supplementary Materials

The following supporting information can be downloaded at: https://www.mdpi.com/article/10.3390/e26090772/s1. Derivations regarding the thermodynamics of transference between phases and opposing predictions for other thermodynamic quantities (entropy and enthalpy of solvation).
