# Supplementary material for: Concentration Scales and Solvation Thermodynamics: Some Theoretical and Experimental Observations Regarding Spontaneity and the Partition Ratio
Source: Entropy (Basel). 2024 Sep 10;26(9):772. doi: 10.3390/e26090772 (PMC11431342; doi:10.3390/e26090772)
Supplement: Supplementary file 1 [file entropy-26-00772-s001.zip › entropy-3107160-supplementary.pdf]

Concentration scales and solvation thermodynamics: some theoretical and experimental observations regarding spontaneity and the partition ratio

Diego J. Raposo da Silva[1,2*], Jéssica I. R. de Souza[1] and Ricardo L. Longo[1,*]

[1)] Departamento de Química Fundamental. Universidade Federal de Pernambuco, 50740-540, Recife, PE, Brazil; ricardo.longo@ufpe.br

[2)] Current address: Escola Politécnica de Pernambuco, Universidade de Pernambuco, Campus Benfica, 50702-001, Recife, PE, Brazil; djrs@poli.br

* Correspondence: djrs@poli.br; Tel.: +55 81 31847500 (DJRS), ricardo.longo@ufpe.br; Tel.: +55 81 2126-8459 (RLL)

Supplementary Materials

Contents

1. Thermodynamics of transference between phases S2

1.1. Standard thermodynamics of transfer applied to solvation S2

1.2. Solvation thermodynamics approach to solvation S9

2. Opposing predictions for other thermodynamic quantities S11

2.1. Solvation entropies S11

2.2. Solvation enthalpies S13

3. References S19

1. Thermodynamics of transference between phases

1.1. Standard thermodynamics of transfer applied to solvation

The freedom of choice of standard states in thermodynamics, and of different concentration scales in the measurements of standard chemical potentials and activity coefficients in solutions, conveniently allowed the measures of thermodynamic properties outside of ideal conditions, such as in ideal gases and diluted solutions. The arbitrariness of choice between these two quantities is due to the fact that the chemical potential per mol[1] of a substance i in a phase α, $\bar{\mu}_{i,\alpha}$, can be decomposed into a standard chemical potential, $\bar{\mu}^{\mathrm{o}}_{i,\alpha}(u)$, which depends on the standard state and the concentration scale u in a given equilibrium temperature T and pressure p, and into another term which contains the activity of the substance in this phase, $a^{(u)}_{i,\alpha}$:

$$\bar{\mu}_{i,\alpha} = \bar{\mu}^{\mathrm{o}}_{i,\alpha}(u) + RT \ln a^{(u)}_{i,\alpha} \tag{A1}$$

with R being the gas constant. The activity in gases, $a^{(p)}_{i,\alpha}$, is given in terms of the fugacity coefficient, $\gamma^{(p)}_{i,\alpha}$, the partial pressure of i in α, $p_{i,\alpha}$, and the standard pressure p^{o}, 100 kPa (recommended) or 1 atm (older literature):

$$a^{(p)}_{i,\alpha} = \gamma^{(p)}_{i,\alpha} p_{i,\alpha}/p^{\mathrm{o}} \tag{A2}$$

Observe that the pressure was expressed as a possible concentration scale for gases, for which the standard state corresponds to a gas with $\bar{\mu}_{i,\alpha} = \bar{\mu}^{\mathrm{o}}_{i,\alpha}(u)$. Such a condition is valid for a hypothetical state with $a^{(p)}_{i,\alpha} = 1$ throughout (see Eq. A1). Ideal gases have $\gamma^{(p)}_{i,\alpha} = 1$, and the hypothetical standard state obeys this condition when $p_{i,\alpha} = p^{\mathrm{o}}$, which is not necessarily valid for real gases because it depends on the chemical nature of the gas and the temperature, amongst other factors. Hence, despite the fact that this standard state is hypothetical, it obeys the same ideal gas behavior as all real systems do under proper conditions, which maintain its practical value.

For liquids, specifically solutions, there is more than one useful standard state. One of them, for the amount of the species i being sufficiently low, is based on the fact that real solutions show a limiting behavior, in a state/phase sometimes called ideal dilute solution[2]. The activity based on such a state is characterized by the activity coefficient $\gamma^{(u)}_{i,\alpha}$, which is equivalent to 1 for ideal dilute solutions, and if the concentration of i is measured according to the scale u, $u_{i,\alpha}$:

$$a^{(u)}_{i,\alpha} = \gamma^{(u)}_{i,\alpha} u_{i,\alpha}/u^{\mathrm{o}} \tag{A3}$$

---

[1] For the chemical potential per molecule, the notation μ will be employed, which is the usual notation for the chemical potential per mol. In this case, however, the symbol $\bar{\mu}$ is used, a distinction based on the use of the symbol $\bar{X}$ for a thermodynamic quantity X per mol of substance. Here, $\bar{\mu} = L\mu$, where L is the Avogadro constant.

[2] If i is highly concentrated, assuming a heterogeneous mixture is not formed, there is another useful standard state based on Raoult's Law.

where u° is the standard concentration, a unit quantity related to such a scale. Mole (or amount) fraction x of i is the ratio of the amount of i in α to the total amount of entities in α, so $u = x$ and then $u^{\circ} = x^{\circ} = 1$. For the amount (or "molar") concentration c, $u_{i,\alpha} = c_{i,\alpha}$ and $u^{\circ} = c^{\circ} = 1$ mol dm^{-3}, whereas the choice of the molality scale implies $u_{i,\alpha} = b_{i,\alpha}$ and $u^{\circ} = b^{\circ} = 1$ mol kg^{-1}. The standard states based on the ideal behaviors are characterized by the condition $\gamma_{i,\alpha}^{(u)} = 1$, even when the solution of i is highly concentrated, such as $u_{i,\alpha} = c^{\circ}$ and $u_{i,\alpha} = b^{\circ}$, or even when there is no solution because α is a pure phase of i, as in the case of the mole fraction standard state, for which $u_{i,\alpha} = x^{\circ} = 1$, or when the concentration is high enough to induce phase separation. Once again, the odd nature of these states does not interfere in their usefulness, because they share the ideal behavior limit with real solutions under proper conditions.

Although the activity and the standard chemical potential do depend on the choices of standard state and concentration scale, the chemical potential *does not*. Thus, the same is true for the difference between the chemical potentials of a species in distinct systems, which is related to the spontaneity of the process of transference of this species between them, or between distinct species at two states of the same system, allowing the inference about the spontaneity of the transition of the system from one state to another, like in the prediction of the likelihood of a chemical reaction. Using a scale u' for i in a phase β and u for i in a phase α, the difference between the chemical potentials of this substance in α e β is given by [1, 2]:

$$\Delta_{\alpha}^{\beta}\bar{G}_i = \bar{\mu}_{i,\beta} - \bar{\mu}_{i,\alpha} = \Delta_{\alpha}^{\beta}\bar{G}_i^{\circ}(u - u') + RT\ln(a_{i,\beta}^{(u')}/a_{i,\alpha}^{(u)}) \tag{A4}$$

with

$$\Delta_{\alpha}^{\beta}G_i^{\circ}(u - u') \coloneqq \bar{\mu}_{i,\beta}^{\circ}(u') - \bar{\mu}_{i,\alpha}^{\circ}(u) \tag{A5}$$

where $\Delta_{\alpha}^{\beta}\bar{G}_i$ corresponds to the Gibbs energy required for the transference of i from one phase to another. Notice that this difference can be used as a measure of the spontaneity of the transference process, and that, when the equilibrium is reached, such transference no longer occurs, at least in a preferable way toward one of the phases, so $\Delta_{\alpha}^{\beta}\bar{G}_i = 0$. Any standard state or concentration scales can be used, and the same difference $\bar{\mu}_{i,\beta} - \bar{\mu}_{i,\alpha}$ would remain. If the transference occurs between a pure gas of i and a solution where the concentration of i is $u_{i,\alpha}$, then under ideal conditions (ideal gas ig and diluted solution in α), $\gamma_{i,\mathrm{ig}}^{(p)} = \gamma_{i,\alpha}^{(u)} = 1$, therefore:

$$\Delta_{\mathrm{ig}}^{\alpha}\bar{G}_i = \Delta_{\mathrm{ig}}^{\alpha}\bar{G}_i^{\circ}(p - u) + RT\ln\left(\frac{u_{i,\alpha}/u^{\circ}}{p_{i,\mathrm{ig}}/p^{\circ}}\right) \tag{A6}$$

Analogously, the transference between the diluted solutions of i in phases α and β could be written as:

$$\Delta_{\alpha}^{\beta}\bar{G}_i = \Delta_{\alpha}^{\beta}\bar{G}_i^{\circ}(u - u) + RT\ln(u_{i,\beta}/u_{i,\alpha}) \tag{A7}$$

considering $u = u'$, that is, measuring the composition in both phases with the same concentration scale.

These relations are valid only for ideal gas and ideal dilute solutions and $\Delta_{\alpha}^{\beta}\bar{G}_i^{\circ}(u - u') = \bar{\mu}_{i,\beta}^{\circ}(u') - \bar{\mu}_{i,\alpha}^{\circ}(u)$ can be used as an estimate of the spontaneity of the process—that is, $\Delta_{\alpha}^{\beta}\bar{G}_i$—if

the term $RT \ln(a_{i,\beta}^{(u')}/a_{i,\alpha}^{(u)})$ in Eq. A4 is considered negligible. That happens when the transference occurs between hypothetical standard states, or similarly, between ideal gases and ideal diluted solutions with $u_{i,\alpha}/u^{\circ} = p_{i,\mathrm{ig}}/p^{\circ}$ (in Eq. A6) or with $u_{i,\beta} = u_{i,\alpha}$[3] (in Eq. A7):

$$\Delta_{\mathrm{ig}}^{\alpha}\bar{G}_i(u_{i,\alpha}/u^{\circ} = p_{i,\mathrm{ig}}/p^{\circ}) = \Delta_{\mathrm{ig}}^{\alpha}\bar{G}_i^{\circ}(p-u) \qquad \text{(A8)}$$

so,

$$\Delta_{\alpha}^{\beta}\bar{G}_i(u_{i,\beta} = u_{i,\alpha}) = \Delta_{\alpha}^{\beta}\bar{G}_i^{\circ}(u-u) \qquad \text{(A9)}$$

The advantage of this approach is due to the fact that $\Delta_{\alpha}^{\beta}\bar{G}_i^{\circ}(u-u')$ can only be determined using the values of $\bar{\mu}_{i,\beta}^{\circ}(u')$ and $\bar{\mu}_{i,\alpha}^{\circ}(u)$, or from the measurements of the equilibrium composition (when $\Delta_{\alpha}^{\beta}\bar{G}_i = 0$), namely

$$\Delta_{\mathrm{ig}}^{\alpha}\bar{G}_i^{\circ}(p-u) = -RT\ln\left(\frac{u_{i,\alpha}/u^{\circ}}{p_{i,\mathrm{ig}}/p^{\circ}}\right)_{\mathrm{eq}} \qquad \text{(A10)}$$

and

$$\Delta_{\alpha}^{\beta}\bar{G}_i^{\circ}(u-u) = -RT\ln(u_{i,\beta}/u_{i,\alpha})_{\mathrm{eq}} \qquad \text{(A11)}$$

The concentration and pressure values are presented with the subscript "eq." to indicate that these values are measured when the equilibrium between phases was already established. Therefore, there is no need to include the activity and fugacity coefficients in the estimation of $\Delta_{\alpha}^{\beta}\bar{G}_i$, and the difference $\bar{\mu}_{i,\beta}^{\circ}(u') - \bar{\mu}_{i,\alpha}^{\circ}(u)$ can also be estimated by Henry constants or the equilibrium solubility of i in gas/solution or solution/solution mixtures, respectively, if both gas and solution in the equilibrium composition can be considered ideal. Additionally, $p_{i,\mathrm{ig}}$, $u_{i,\beta}$ or $u_{i,\alpha}$ (in Eqs. A6 and A7) influence the spontaneity of the transference of i between phases, if the conditions for the validity of the approximations $\Delta_{\mathrm{ig}}^{\alpha}\bar{G}_i \cong \Delta_{\mathrm{ig}}^{\alpha}\bar{G}_i^{\circ}$ (or $\Delta_{\alpha}^{\beta}\bar{G}_i \cong \Delta_{\alpha}^{\beta}\bar{G}_i^{\circ}$) hold true.

On the other hand, this approach is quite limited because only very diluted solutions with concentrations small enough to be equivalent to those of the ideal gas, in such a way that $u_{i,\alpha}/u^{\circ} = p_{i,\mathrm{ig}}/p^{\circ}$)[4], can be used, and investigations about transference between phases with $u_{i,\beta} = u_{i,\alpha}$ is limited to cases where only the effects of external factors, such as the ionic strength, on the process can be addressed. The approach is characterized by another problem, namely the loss of the independency of $\Delta_{\alpha}^{\beta}\bar{G}_i$ with the concentration scale because $\Delta_{\alpha}^{\beta}\bar{G}_i^{\circ}(u-u')$ depends on the choice of both u and u'. It will be shown that this disadvantage is particularly troublesome for the definition of the solvation energy, leading to inconsistent and contradictory predictions and conclusions.

---

[3] The equivalence among the interpretations, i.e., transference between hypothetical states ≡ transference between real states where the equalities are valid, is present because the standard state behaves as ideal—not just at the standard pressure (for gases) or concentration (dilute solutions)—but at any pressure or concentration for any substance at any temperature. That includes when $u_{i,\alpha}/u^{\circ} = p_{i,\alpha}/p^{\circ}$ or $u_{i,\beta} = u_{i,\alpha}$ between pairs of hypothetical states.

[4] For instance, the amount (or "molar") concentration of an ideal gas at 100 kPa is 0.0409 mol/L.

At the beginning of the 20th century, researchers began to suspect a relationship between the solvation energy (or the amount of work that is necessary or produced by the dissolution of a substance into another) and the transference of a substance from ideal gas (where the interactions between particles are negligible) to a pure liquid or mixture. Hence, by using the gas/solution equilibrium, one could measure the amounts of a substance in both phases, and the higher the amount of the substance in one phase, the more soluble it was in that solvent. The problem is that the "amount of substance" may be defined in different ways. For molecules of type i in ideal gas, such an amount can either be measured by its partial pressure, $p_{i,\mathrm{ig}}$, or its ("molar") concentration, $c_{i,\mathrm{ig}}$; and the amount of solute molecules in a solvent is either related to its mole fraction, $x_{i,\mathrm{s}}$, or to its ("molar") concentration, $c_{i,\mathrm{s}}$, or molal concentration, $b_{i,\mathrm{s}}$, amongst other concentration scales. Therefore, if the amount of i in an ideal gas is quantified by its pressure in a solution, through one of these concentration scales (x, c, ou b), then, the following expressions for the standard Gibbs energies of solvation[5] (as consequences of Eq. A10) are obtained:

$$\Delta_{\mathrm{ig}}^{\mathrm{s}}\bar{G}_i^{\mathrm{o}}(p-x) = -RT\ln\left(\frac{x_{i,\mathrm{s}}/x^{\mathrm{o}}}{p_{i,\mathrm{ig}}/p^{\mathrm{o}}}\right)_{\mathrm{eq}} \quad \text{(A12)}$$

$$\Delta_{\mathrm{ig}}^{\mathrm{s}}\bar{G}_i^{\mathrm{o}}(p-c) = -RT\ln\left(\frac{c_{i,\mathrm{s}}/c^{\mathrm{o}}}{p_{i,\mathrm{ig}}/p^{\mathrm{o}}}\right)_{\mathrm{eq}} \quad \text{(A13)}$$

$$\Delta_{\mathrm{ig}}^{\mathrm{s}}\bar{G}_i^{\mathrm{o}}(p-b) = -RT\ln\left(\frac{b_{i,\mathrm{s}}/b^{\mathrm{o}}}{p_{i,\mathrm{ig}}/p^{\mathrm{o}}}\right)_{\mathrm{eq}} \quad \text{(A14)}$$

In the amount ("molar") concentration scale for both gas and liquid phase, the standard Gibbs solvation energy is:

$$\Delta_{\mathrm{ig}}^{\mathrm{s}}\bar{G}_i^{\mathrm{o}}(c-c) = -RT\ln(c_{i,\mathrm{s}}/c_{i,\mathrm{ig}})_{\mathrm{eq}} \quad \text{(A15)}$$

Each one of these Gibbs energies is related to hypothetical transfer processes where i goes from ideal gas (ig) to the solution (s). These are hypothetical because the standard phases (standard state) behave unrealistically, but the transfer energies for these processes coincide with the transfer between real ideal gases and diluted solutions. Therefore, these energies can only be used, in principle, under these conditions. The transfer processes are described according to the following Gibbs energies, and through the scheme in Figure S1:

1) $\Delta_{\mathrm{ig}}^{\mathrm{s}}\bar{G}_i^{\mathrm{o}}(p-x)$: transfer of 1 mol of hypothetical gas i—which behaves as ideal gas—obeying $p_{i,\mathrm{ig}} = c_{i,\mathrm{ig}}RT$ for a partial pressure $p_{i,\mathrm{ig}}$ and amount ("molar") concentration $c_{i,\mathrm{ig}}$ of i, at standard pressure p^{o} at any temperature T—to a hypothetical solution of i in s—which behaves as an ideal solution even when $x_{i,\mathrm{s}} = x^{\mathrm{o}} = 1$, where there is no solvent whatsoever, only the solute i; therefore, it is a solution only in the theoretical sense: it is defined to be;

2) $\Delta_{\mathrm{ig}}^{\mathrm{s}}\bar{G}_i^{\mathrm{o}}(p-c)$: transfer of 1 mol of hypothetical gas i—which behaves as ideal gas at standard pressure p^{o} and at any temperature T—to a hypothetical solution of i in

[5] Observe that the notation of the liquid phase α was replaced by the representation of the solvent s. The notation for a general phase is α, namely fluids with an arbitrary number of components.

s—which behaves as ideal solution even when $c_{i,\mathrm{s}} = c^{\mathrm{o}} = 1\ \mathrm{mol\ L^{-1}}$; therefore, it is a theoretical solution because it is highly concentrated and yet behaves as ideal diluted solution;

3) $\Delta_{\mathrm{ig}}^{\mathrm{s}}\bar{G}_i^{\mathrm{o}}(p-b)$: transfer of 1 mol of hypothetical gas i—which behaves as ideal gas at standard pressure p^{o} and at any temperature T—to a hypothetical solution of i in s—which behaves as an ideal solution even when $b_{i,\mathrm{s}} = b^{\mathrm{o}} = 1\ \mathrm{mol\ kg^{-1}}$, again, a high concentration solution;

4) $\Delta_{\mathrm{ig}}^{\mathrm{s}}\bar{G}_i^{\mathrm{o}}(c-c)$: transfer of 1 mol of an hypothetical gas i—which behaves as ideal gas at pressure $p^{\square} = c^{\mathrm{o}}RT$ and at any temperature T—to a hypothetical solution of i in s—which behaves as ideal solution even when $c_{i,\mathrm{s}} = c^{\mathrm{o}} = 1\ \mathrm{mol\ L^{-1}}$, i.e., a concentrated solution with a diluted solution behavior.

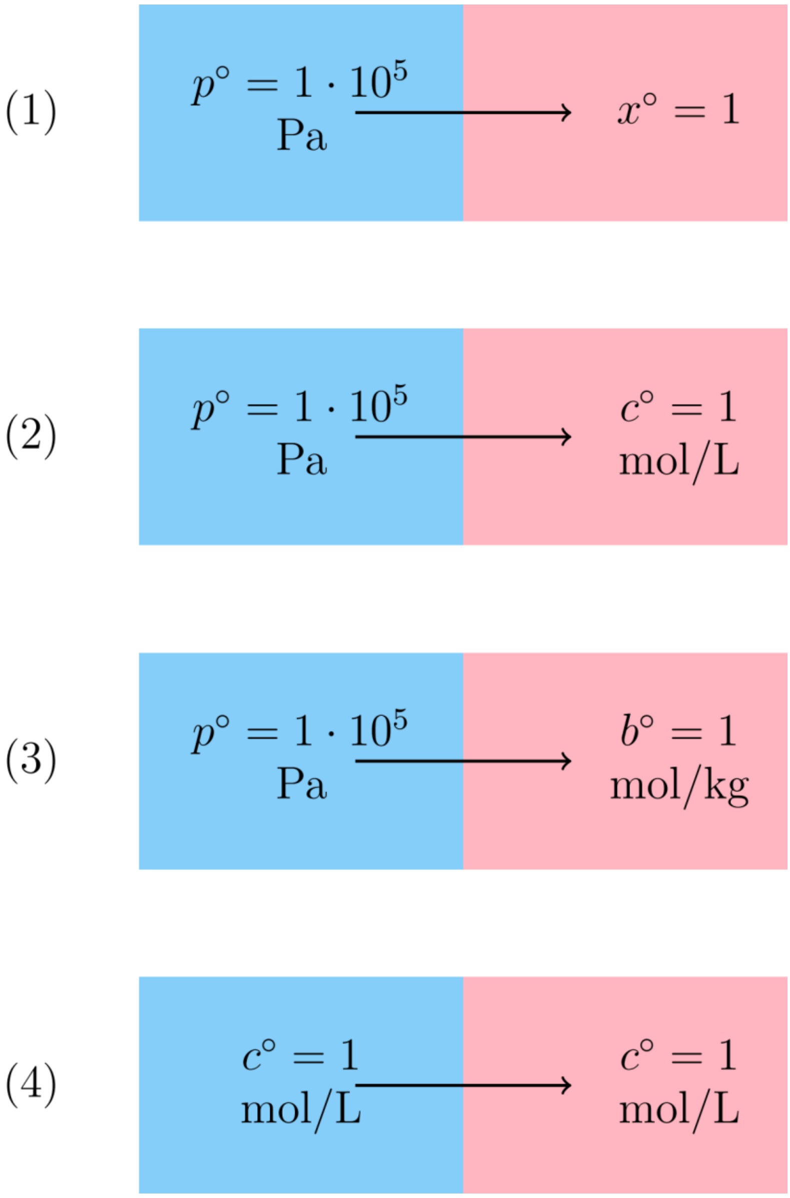

Figure S1: Schematic representation of the transfer processes between hypothetical phases and the respective standard Gibbs energies, calculated according to Eqs. A12 to A14.

One of the most intriguing discussions concerning the study of solvation involves which concentration scale or which of these standard Gibbs energies provides proper and adequate information about the solvation. Indeed, it can be shown that these lead to different energies, *even with opposite signs*, and if all hypothetical states in the solution behave ideally, i.e., ideal dilute solutions, these Gibbs energies are all related, from Eqs. A12 to A14, as

$$\Delta_{\mathrm{ig}}^{\mathrm{s}}\bar{G}_i^{\mathrm{o}}(p-c) = -RT\ln\left(\frac{c_{i,\mathrm{s}}/c^{\mathrm{o}}}{p_{i,\mathrm{ig}}/p^{\mathrm{o}}}\right)_{\mathrm{eq}} = -RT\ln\left(\frac{c_{i,\mathrm{s}}/c^{\mathrm{o}}}{c_{i,\mathrm{ig}}RT/p^{\mathrm{o}}}\right)_{\mathrm{eq}} = -RT\ln\left(\frac{c_{i,\mathrm{s}}}{c_{i,\mathrm{ig}}}\right)_{\mathrm{eq}} - RT\ln\left(\frac{p^{\mathrm{o}}}{c^{\mathrm{o}}RT}\right)$$

$$\Delta_{\mathrm{ig}}^{\mathrm{s}}\bar{G}_i^{\mathrm{o}}(c-c) = \Delta_{\mathrm{ig}}^{\mathrm{s}}\bar{G}_i^{\mathrm{o}}(p-c) - RT\ln(c^{\mathrm{o}}RT/p^{\mathrm{o}}) \tag{A16}$$

The constant $RT\ln(c^{\mathrm{o}}RT/p^{\mathrm{o}})$ is equal to 1.89 kcal/mol for $p^{\mathrm{o}} = 1$ atm and $T = 298.15$ K, and converts the Gibbs energy of the ideal gas at 1 atm as the standard state to the ideal gas at 1 mol/L, keeping the solution standard state based on the amount ("molar") concentration scale. This term has caused a lot of confusion and discussion for those who calculated the pK_{a}, which depends on solvation energies, using computational methods, because it was not until much later that it was understood that the same standard state needed to be kept for the solute in the gas and in the solution, that is, in the same ("molar") concentration, for reasons to be clarified later, a direct result of ST.

The concentration scales can be related to each other when the solution is diluted. The mole fraction of a substance i in a solvent s, $x_{i,\mathrm{s}}$, is related to its ("molar") concentration $c_{i,\mathrm{s}}$ and to its molarity $b_{i,\mathrm{s}}$ as:

$$x_{i,\mathrm{s}} = M_{\mathrm{s}}c_{i,\mathrm{s}}/d_{\mathrm{s}} = M_{\mathrm{s}}b_{i,\mathrm{s}} \tag{A17}$$

where M_{s} and d_{s} are the molar mass and mass density of the solvent. Therefore, we can relate $\Delta_{\mathrm{ig}}^{\mathrm{s}}\bar{G}_i^{\mathrm{o}}(p-x)$, $\Delta_{\mathrm{ig}}^{\mathrm{s}}\bar{G}_i^{\mathrm{o}}(p-c)$, $\Delta_{\mathrm{ig}}^{\mathrm{s}}\bar{G}_i^{\mathrm{o}}(p-b)$, and $\Delta_{\mathrm{ig}}^{\mathrm{s}}\bar{G}_i^{\mathrm{o}}(c-c)$ accordingly:

$$\Delta_{\mathrm{ig}}^{\mathrm{s}}\bar{G}_i^{\mathrm{o}}(p-x) = -RT\ln\left(\frac{x_{i,\mathrm{s}}/x^{\mathrm{o}}}{p_{i,\mathrm{ig}}/p^{\mathrm{o}}}\right)_{\mathrm{eq}} = -RT\ln\left(\frac{c_{i,\mathrm{s}}/c^{\mathrm{o}}}{p_{i,\mathrm{ig}}/p^{\mathrm{o}}}\right)_{\mathrm{eq}} - RT\ln\left(\frac{c^{\mathrm{o}}M_{\mathrm{s}}}{x^{\mathrm{o}}d_{\mathrm{s}}}\right)$$

$$\Delta_{\mathrm{ig}}^{\mathrm{s}}\bar{G}_i^{\mathrm{o}}(p-x) = -RT\ln\left(\frac{x_{i,\mathrm{s}}/x^{\mathrm{o}}}{p_{i,\mathrm{ig}}/p^{\mathrm{o}}}\right)_{\mathrm{eq}} = -RT\ln\left(\frac{b_{i,\mathrm{s}}/b^{\mathrm{o}}}{p_{i,\mathrm{ig}}/p^{\mathrm{o}}}\right)_{\mathrm{eq}} - RT\ln\left(\frac{b^{\mathrm{o}}M_{\mathrm{s}}}{x^{\mathrm{o}}}\right)$$

$$\begin{aligned}\Delta_{\mathrm{ig}}^{\mathrm{s}}\bar{G}_i^{\mathrm{o}}(p-x) &= \Delta_{\mathrm{ig}}^{\mathrm{s}}\bar{G}_i^{\mathrm{o}}(p-c) - RT\ln\left(\frac{c^{\mathrm{o}}M_{\mathrm{s}}}{x^{\mathrm{o}}d_{\mathrm{s}}}\right) = \Delta_{\mathrm{ig}}^{\mathrm{s}}\bar{G}_i^{\mathrm{o}}(p-b) - RT\ln(b^{\mathrm{o}}M_{\mathrm{s}}/x^{\mathrm{o}}) \\ &= \Delta_{\mathrm{ig}}^{\mathrm{s}}\bar{G}_i^{\mathrm{o}}(c-c) - RT\ln\left(\frac{M_{\mathrm{s}}p^{\mathrm{o}}}{x^{\mathrm{o}}RTd_{\mathrm{s}}}\right)\end{aligned} \tag{A18}$$

The last equality can be written in terms of the standard state ideal gas volume, $\bar{V}_{\mathrm{ig}} = p^{\mathrm{o}}/RT$, and the solvent molar volume, $\bar{V}_{\mathrm{s}} = d_{\mathrm{s}}/M_{\mathrm{s}}$, as

$$\Delta_{\mathrm{ig}}^{\mathrm{s}}\bar{G}_i^{\mathrm{o}}(p-x) = \Delta_{\mathrm{ig}}^{\mathrm{s}}\bar{G}_i^{\mathrm{o}}(c-c) - RT\ln(\bar{V}_{\mathrm{ig}}/\bar{V}_{\mathrm{s}}) \tag{A19}$$

The unavoidable conclusion from these equations is that there are numerical differences between the SSGEs in different concentration scales, and these differences depend on the solvent (e.g., mass density and molar mass), and consequently, do not play any role in the solute–solvent interactions (for it does not depend on i). As shown by A. Ben-Naim, the processes described by Eqs. A12–A14 (the first one called process-x, and the third, process-b) include an interaction contribution and other terms, related to the translational freedom of the solute in different phases [3], or with the change in volume experienced by the solute when it migrates from the gas to the solution [2]. On the other hand, $\Delta_{\mathrm{ig}}^{\mathrm{s}}\bar{G}_i^{\mathrm{o}}(c-c)$, often called process-$c$, lacks these terms and it is possible to prove its intimate and exclusive relationship with the interactions between the transferred molecule (the solute) and the molecules in the liquid (solute and solvent), because the interactions in ideal gas are negligible.

1.2 – Solvation thermodynamics approach to solvation

The microscopic description of solvation introduced by Ben-Naim, but also explored by others (e.g., Widom, etc.), provided tools to estimate the energies related to the solvation process. One of the most useful and relevant demonstrations is the SGE of i in α, $\Delta G_{i,\alpha}^{*}$, which coincides with the energy associated with process-c, namely

$$\Delta_{\mathrm{ig}}^{\alpha}\bar{G}_i^{\mathrm{o}}(c-c) = \Delta\bar{G}_{i,\alpha}^{*} \tag{A20}$$

However, one can also show that this is not the case for other thermodynamics state functions, such as entropy and enthalpy, when applied to solvation. Naturally, the same holds for these functions in other processes (x and b). To demonstrate Eq. A20, it is important to follow the steps proposed by Ben-Naim. The Gibbs energy of a process is given by the difference between the chemical potentials of species i in the final and initial state/phase α. The chemical potential of i in α, $\mu_{i,\alpha}$, is composed of a term related to the work required to insert the particle (or its rigid conformer for a general case) in such a phase in a fixed position and orientation, the pseudo-chemical potential (PCP), $\mu_{i,\alpha}^{*}$, other term corresponding to the energy required to give momentum to this molecule, $kT\ln\Lambda_i^3$, and yet another one due to the amount of i in phase α, $kT\ln\rho_{i,\alpha}$, where $\rho_{i,\alpha}$ is the number density of i in α and k is the Boltzmann constant:

$$\mu_{i,\alpha} = \mu_{i,\alpha}^{*} + kT\ln\Lambda_i^3 + kT\ln\rho_{i,\alpha} \tag{A21}$$

The constant Λ_i^3 only depends on the mass of the particle i, of the temperature and fundamental constants. Notice that only one term is related to solute–solvent interactions in this expression, the PCP. Multiplying Eq. A21 by Avogadro constant, L, and adding $RT\ln L$ on both sides of the equation, the chemical potential per mol of i is:

$$\bar{\mu}_{i,\alpha} = \bar{\mu}_{i,\alpha}^{*} + RT\ln(L\Lambda_i^3) + RT\ln c_{i,\alpha} \tag{A22}$$

where the relations $\bar{\mu}_{i,\alpha} = L\mu_{i,\alpha}$, $\bar{\mu}_{i,\alpha}^{*} = L\mu_{i,\alpha}^{*}$, $R = Lk$, and $\rho_{i,\alpha} = Lc_{i,\alpha}$ were applied. The difference between the chemical potential of i in α and i in the ideal gas (ig) leads to the Gibbs energy related to the transfer of the particle from one phase to the other (per mol of i):

$$\Delta_{\mathrm{ig}}^{\alpha}\bar{G}_i = \bar{\mu}_{i,\alpha} - \bar{\mu}_{i,\mathrm{ig}} = \bar{\mu}_{i,\alpha}^{*} - \bar{\mu}_{i,\mathrm{ig}}^{*} + RT\ln(c_{i,\alpha}/c_{i,\mathrm{ig}}) \tag{A23}$$

Since *the only terms related to interactions between i with the remaining molecules (either in α or in ig) are the PCPs*, it is natural to define the solvation Gibbs energy (SGE) as the difference $\bar{\mu}^*_{i,\alpha} - \bar{\mu}^*_{i,\mathrm{ig}}$, which lacks energetic terms of any other kind:

$$\Delta\bar{G}^*_{i,\alpha} \coloneqq \bar{\mu}^*_{i,\alpha} - \bar{\mu}^*_{i,\mathrm{ig}} \tag{A24}$$

where $\Delta\bar{G}^*_{i,\alpha}$ is the SGE per mol of i, being related to Eq. 1 (due to the solvation a single molecule) by the factor L, i.e., $\Delta\bar{G}^*_{i,\alpha} = L\Delta G^*_{i,\alpha}$. Therefore, *the procedure adopted by Ben-Naim was to detail the different energetic contributions in the transfer Gibbs energy of i from a phase where it does not interact with the surrounding particles (the ideal gas) to a solution, phase α, and solely selects the ones related to solute–solvent interactions.* At equilibrium, $\Delta^{\alpha}_{\mathrm{ig}}\bar{G}_i = 0$ implies, through Eqs. A23 and A24, that $\Delta\bar{G}^*_{i,\alpha} = -RT\ln(c_{i,\alpha}/c_{i,\mathrm{ig}})_{\mathrm{eq}}$. Because it is also true that $\Delta^{\alpha}_{\mathrm{ig}}\bar{G}^{\mathrm{o}}_i(c-c) = -RT\ln(c_{i,\alpha}/c_{i,\mathrm{ig}})_{\mathrm{eq}}$ (Eq. A15), it is promptly demonstrated that $\Delta\bar{G}^*_{i,\alpha} = \Delta^{\alpha}_{\mathrm{ig}}\bar{G}^{\mathrm{o}}_i(c-c)$ (Eq. A20). However, in general, the process-c differs from the one described by $\Delta\bar{G}^*_{i,\alpha}$. The function $\Delta^{\alpha}_{\mathrm{ig}}\bar{G}^{\mathrm{o}}_i(c-c)$ describes the transfer between hypothetical standard phases (in gas and solution), which behave as ideal (and, therefore, have an interpretation limited to these conditions) and terms both related and unrelated to interactions, such as the momentum term of the molecule, $RT\ln(L\Lambda_i^3)$. The subtraction between the chemical potentials leads to a cancelation of this second part, and the Gibbs energies $\Delta^{\alpha}_{\mathrm{ig}}\bar{G}^{\mathrm{o}}_i(c-c)$ and $\Delta\bar{G}^*_{i,\alpha}$ are numerically equivalent. The transfer process of the SGE, however, is based on a strict and precisely defined procedure: the transference of i from an ideal gas to α, in a specific position and orientation in each phase. *The momentum term of the molecule, therefore, is excluded by default in the definition of the solvation*. And, it does not only apply to diluted solutions, but to any composition. The distinction between process-c and the solvation process according to the ST is most directly seen in the solvation entropies, which unlike the SGEs, are not the same (next section).

Another way to verify the identity in Eq. A20 is to recall that $\Delta^{\alpha}_{\mathrm{ig}}\bar{G}^{\mathrm{o}}_i(c-c)$ is the Gibbs energy $\Delta^{\alpha}_{\mathrm{ig}}\bar{G}_i$ when the same standard concentration is chosen for both phases, i.e., $c_{i,\alpha} = c_{i,\mathrm{ig}}$, and the phases behave ideally, so $\Delta^{\alpha}_{\mathrm{ig}}\bar{G}_i(c_{i,\alpha} = c_{i,\mathrm{ig}}) = \Delta^{\alpha}_{\mathrm{ig}}\bar{G}^{\mathrm{o}}_i(c-c) = \Delta\bar{G}^*_{i,\alpha}$ using Eqs. A23 and A24.

Ben-Naim has rigorously demonstrated that process-c provides the correct, that is, only solvation-related Gibbs energy, but he has not shown the experimental validity of this result. That is, given his proposal for defining the SGE—based on statistical mechanics principles and in the relation between solute and solvent interactions and the solvation process—he did prove how to use it and understand it.

2. Opposing predictions for other thermodynamic quantities

2.1. Solvation entropies

By following the same procedure of chemical potential separation in terms of solvent–solute interactions and the remaining contributions, and then defining the solvation properly as only the first contribution, it is useful to start by using the relation between entropy and chemical potential (at constant pressure p and number of molecules j, N_j):

$$\bar{S}_{i,\alpha} = -\left(\frac{\partial \bar{\mu}_{i,\alpha}}{\partial T}\right)_{p,N_j} \tag{B1}$$

From Eq. A22, it is possible to show that:

$$\bar{S}_{i,\alpha} = -\left(\frac{\partial \bar{\mu}^*_{i,\alpha}}{\partial T}\right)_{p,N_j} - R\ln(L\Lambda_i^3) - RT\left[\frac{\partial \ln(L\Lambda_i^3)}{\partial T}\right]_{p,N_j} - R\ln c_{i,\alpha} - RT\left[\frac{\partial \ln c_{i,\alpha}}{\partial T}\right]_{p,N_j}$$

$$\bar{S}_{i,\alpha} = -\left(\frac{\partial \bar{\mu}^*_{i,\alpha}}{\partial T}\right)_{p,N_j} - R\ln(L\Lambda_i^3) - \frac{RT}{L\Lambda_i^3}\left[\frac{\partial (L\Lambda_i^3)}{\partial T}\right]_{p,N_j} - R\ln c_{i,\alpha} + RT\left[\frac{\partial \ln V_\alpha}{\partial T}\right]_{p,N_j}$$

$$\bar{S}_{i,\alpha} = -\left(\frac{\partial \bar{\mu}^*_{i,\alpha}}{\partial T}\right)_{p,N_j} - R\ln(c_{i,\alpha}L\Lambda_i^3) - \frac{RT}{\Lambda_i^3}\left[\frac{\partial \Lambda_i^3}{\partial T}\right]_{p,N_j} + RT\frac{1}{V_\alpha}\left[\frac{\partial V_\alpha}{\partial T}\right]_{p,N_j}$$

$$\bar{S}_{i,\alpha} = -\left(\frac{\partial \bar{\mu}^*_{i,\alpha}}{\partial T}\right)_{p,N_j} - R\ln(c_{i,\alpha}L\Lambda_i^3) - \frac{RT}{\Lambda_i^3}\left[\frac{\partial \Lambda_i^3}{\partial T}\right]_{p,N_j} + \gamma_\alpha RT \tag{B2}$$

where γ_α is the thermal expansion coefficient (at constant pressure) of α, equivalent to $V_\alpha^{-1}[\partial V_\alpha/\partial T]_{p,N_j}$. The difference between the entropies of i in α and in ideal gas becomes

$$\Delta_{\text{ig}}^{\alpha}\bar{S}_i = -\left(\frac{\partial \bar{\mu}^*_{i,\alpha}}{\partial T}\right)_{p,N_j} + \left(\frac{\partial \bar{\mu}^*_{i,\text{ig}}}{\partial T}\right)_{p,N_j} - R\ln(c_{i,\alpha}/c_{i,\text{ig}}) + (\gamma_\alpha - \gamma_{\text{ig}})RT \tag{B3}$$

It is clear which part of the entropy is related to the solvation, namely the term based on solute–solvent interaction changes with the temperature:

$$\Delta\bar{S}^*_{i,\alpha} \coloneqq -\left(\frac{\partial \bar{\mu}^*_{i,\alpha}}{\partial T}\right)_{p,N_j} + \left(\frac{\partial \bar{\mu}^*_{i,\text{ig}}}{\partial T}\right)_{p,N_j} = -\left[\frac{\partial\left(\bar{\mu}^*_{i,\alpha} - \bar{\mu}^*_{i,\text{ig}}\right)}{\partial T}\right]_{p,N_j} = -\left(\frac{\partial \Delta\bar{G}^*_{i,\alpha}}{\partial T}\right)_{p,N_j} \tag{B4}$$

To relate this entropy with the equivalent quantity according to process-c, all that is needed is to ascertain which value of $\Delta_{\text{ig}}^{\alpha}\bar{S}_i$ corresponds to the condition $c_{i,\alpha} = c_{i,\text{ig}}$. Therefore, from Eqs. B3 and B4:

$$\Delta_{\text{ig}}^{\alpha}\bar{S}_i(c_{i,\alpha} = c_{i,\text{ig}}) \equiv \Delta_{\text{ig}}^{\alpha}\bar{S}_i^{\text{o}}(c-c) = \Delta\bar{S}^*_{i,\alpha} + (\gamma_\alpha - \gamma_{\text{ig}})RT \tag{B5}$$

and because $\gamma_{\text{ig}} = 1/T$:

$$\Delta_{\text{ig}}^{\alpha}\bar{S}_i^{\text{o}}(c-c) = \Delta\bar{S}^*_{i,\alpha} + \gamma_\alpha RT - R \tag{B6}$$

It must be emphasized that the relations in Eqs. A20 and B6 only hold because these are assumed to be ideal hypothetical states in ideal gas and solution, allowing the association of often used standard quantities and the ST thermodynamic variables. The values of $\Delta\bar{G}^*_{i,\alpha}$ and $\Delta\bar{S}^*_{i,\alpha}$, however, are not restricted to this condition, and are related to the solvation process even in concentrated solutions from non-ideal gases. It is also important to notice that, although the Gibbs energy in process-c and $\Delta\bar{G}^*_{i,\alpha}$ are numerically equivalent at equilibrium (Eq. A20), this equivalence does not hold for the entropy, because $\Delta\bar{S}^*_{i,\alpha} \neq \Delta_{\text{ig}}^{\alpha}\bar{S}_i^{\text{o}}(c-c)$ (Eq. B6).

The relationship between $\Delta\bar{S}^*_{i,\alpha}$ and $\Delta_{\text{ig}}^{\alpha}\bar{S}_i^{\text{o}}(p-x)$ requires the conversion of $c_{i,\text{s}}$ into $x_{i,\text{s}}$ in Eq. B3 (now specifying that phase α only contains the solvent s), and the ideal gas state equation:

$$\Delta_{\text{ig}}^{\text{s}}\bar{S}_i = \Delta\bar{S}^*_{i,\text{s}} - R\ln(c_{i,\alpha}/c_{i,\text{ig}}) + \gamma_\alpha RT - R = \Delta\bar{S}^*_{i,\text{s}} - R\ln\left(\frac{RTd_\text{s}x_{i,\alpha}}{M_\text{s}p_{i,\text{ig}}}\right) + \gamma_\alpha RT - R$$

Because the transference of i happens between standard phases, from a hypothetical ideal gas with $p_{i,\mathrm{ig}} = p^{\mathrm{o}}$ for a solution with $x_{i,\alpha} = x^{\mathrm{o}} = 1$, then

$$\Delta_{\mathrm{ig}}^{\mathrm{s}}\bar{S}_i(x_{i,\alpha} = 1; p_{i,\mathrm{ig}} = p^{\mathrm{o}}) = \Delta_{\mathrm{ig}}^{\mathrm{s}}\bar{S}_i^{\mathrm{o}}(p - x) = \Delta\bar{S}_{i,\mathrm{s}}^{*} - R\ln\left(\frac{RTd_{\mathrm{s}}}{M_{\mathrm{s}}p^{\mathrm{o}}}\right) + \gamma_\alpha RT - R$$

$$\Delta_{\mathrm{ig}}^{\mathrm{s}}\bar{S}_i^{\mathrm{o}}(p - x) = \Delta\bar{S}_{i,\mathrm{s}}^{*} - R\ln(\bar{V}_{\mathrm{ig}}/\bar{V}_{\mathrm{s}}) + \gamma_\alpha RT - R \qquad \text{(B7)}$$

The conversion term from process-x to the ST entropy, specifically in water (w), with the thermal expansion coefficient of 0.265 °C^{-1}, that is, $\gamma_{\mathrm{w}} = 1/(0.265\ ^{\circ}\mathrm{C}) = 1/[(0.265 + 273.15)\ \mathrm{K}] = 3.657 \times 10^{-3}\ \mathrm{K}^{-1}$ [4]:

$$\begin{aligned}-R\ln(\bar{V}_{\mathrm{ig}}/\bar{V}_{\mathrm{s}}) + \gamma_\alpha RT - R &= R\left[\ln(\bar{V}_{\mathrm{s}}/\bar{V}_{\mathrm{ig}}) + \gamma_\alpha T - 1\right] \\ &= 1.987\ \mathrm{cal\ K^{-1}mol^{-1}}\left[\ln\left(\frac{0.01807}{24.4655}\right) + 3.657 \times 10^{-3}\ \mathrm{K}^{-1} \times 298.15\ \mathrm{K}^{-1} - 1\right] \\ &= -14.15\ \mathrm{cal\ K^{-1}\ mol^{-1}}\end{aligned}$$

$$\Delta\bar{S}_{i,\mathrm{w}}^{*} = \Delta_{\mathrm{ig}}^{\mathrm{w}}\bar{S}_i^{\mathrm{o}}(p - x) + 14.15\ \mathrm{cal\ K^{-1}\ mol^{-1}} \qquad \text{(B8)}$$

Therefore, if $-14.15\ \mathrm{cal\ K^{-1}\ mol^{-1}} < \Delta_{\mathrm{ig}}^{\mathrm{w}}\bar{S}_i^{\mathrm{o}}(p - x) < 0\ \mathrm{cal\ K^{-1}\ mol^{-1}}$ for a substance i in water, the signs of $\Delta\bar{S}_{i,\mathrm{w}}^{*}$ and $\Delta_{\mathrm{ig}}^{\mathrm{w}}\bar{S}_i^{\mathrm{o}}(p - x)$ will be opposite.

For contradictory predictions with a heterogeneous mixture of i between two imiscible solvents α and β, according to Eq. B5:

$$\Delta_{\mathrm{ig}}^{\beta}\bar{S}_i^{\mathrm{o}}(p - x) - \Delta_{\mathrm{ig}}^{\alpha}\bar{S}_i^{\mathrm{o}}(p - x) = \Delta\bar{S}_{i,\beta}^{*} - \Delta\bar{S}_{i,\alpha}^{*} - R\ln(\bar{V}_\alpha/\bar{V}_\beta) + (\gamma_\beta - \gamma_\alpha)RT$$

$$\Delta_{\alpha}^{\beta}\bar{S}_i^{\mathrm{o}}(x - x) = \Delta_{\alpha}^{\beta}\bar{S}_i^{*} - R\ln(\bar{V}_\alpha/\bar{V}_\beta) + (\gamma_\beta - \gamma_\alpha)RT \qquad \text{(B9)}$$

So, to investigate the example of water/hexadecane mixtures with a thermal expansion coefficient of hexadecane of $\gamma_{\mathrm{h}} = 0.891 \times 10^{-3}\ \mathrm{K}^{-1}$ at 100 kPa:

$$\begin{aligned}-R\ln(\bar{V}_{\mathrm{w}}/\bar{V}_{\mathrm{h}}) + (\gamma_{\mathrm{h}} - \gamma_{\mathrm{w}})RT &= R[-\ln(\bar{V}_{\mathrm{w}}/\bar{V}_{\mathrm{h}}) + (\gamma_{\mathrm{h}} - \gamma_{\mathrm{w}})T] \\ &= 1.987\ \mathrm{cal\ K^{-1}\ mol^{-1}}\left[-\ln\left(\frac{0.01807}{0.2940}\right)\right. \\ &\quad \left. + (0.891 - 3.657) \times 10^{-3}\ \mathrm{K}^{-1} \times 298.15\ \mathrm{K}\right] = 3.903\ \mathrm{cal\ K^{-1}\ mol^{-1}}\end{aligned}$$

$$\Delta_{\mathrm{w}}^{\mathrm{h}}\bar{S}_i^{*} = \Delta_{\mathrm{w}}^{\mathrm{h}}\bar{S}_i^{\mathrm{o}}(x - x) - 3.903\ \mathrm{cal\ K^{-1}\ mol^{-1}} \qquad \text{(B10)}$$

Thus, for substances with standard solvation entropies $\Delta_{\mathrm{w}}^{\mathrm{h}}\bar{S}_i^{\mathrm{o}}(x - x)$ that are positive and between 0 and 3.903 cal K^{-1} mol^{-1}, the sign of $\Delta_{\mathrm{w}}^{\mathrm{h}}\bar{S}_i^{*}$ will be negative. From the substances listed in Table 1 of the paper by Abraham et al. [5], almost all values of $\Delta_{\mathrm{w}}^{\mathrm{h}}\bar{S}_i^{\mathrm{o}}(x - x)$ are positive, and among these, all are superior to 3.903 cal K^{-1} mol^{-1}, preventing the contradiction between the predictions caused by the incorrect choice of standard states. However, substances such as benzaldehyde, with $\Delta_{\mathrm{w}}^{\mathrm{h}}\bar{S}_i^{\mathrm{o}}(x - x) = 13.5\ \mathrm{cal\ K^{-1}\ mol^{-1}}$, aniline (15.0 cal K^{-1} mol^{-1}), nitromethane (9.0 cal K^{-1} mol^{-1}), acetonitrile (12.4 cal K^{-1} mol^{-1}) and benzil chloride (12.5 cal K^{-1} mol^{-1}) have transfer entropies two or even three times bigger than 3.9 cal K^{-1} mol^{-1}. These cases might be prone to a comparison between the predictions of $\Delta_{\mathrm{w}}^{\mathrm{h}}\bar{S}_i^{\mathrm{o}}(x - x)$ e $\Delta_{\mathrm{w}}^{\mathrm{h}}\bar{S}_i^{*}$ ($\neq \Delta_{\mathrm{w}}^{\mathrm{h}}\bar{S}_i^{\mathrm{o}}(c - c)$, important to recall) and the experimental confirmation of one of them.

2.2. Solvation enthalpies

According to the procedure developed for Gibbs energy and entropy, the enthalpy changes, using Eqs. A19, A20 and B7, can be expressed as:

$$\Delta\bar{H}^*_{i,\alpha} = \Delta\bar{G}^*_{i,\alpha} + T\Delta\bar{S}^*_{i,\alpha} = \Delta\bar{G}^*_{i,\alpha} + T\left(\frac{\partial\Delta\bar{G}^*_{i,\alpha}}{\partial T}\right)_{p,N_j}$$

$$\Delta\bar{H}^*_{i,\alpha} = \Delta^{\alpha}_{\mathrm{ig}}\bar{G}^{\mathrm{o}}_i(p-x) - RT\ln\left(\frac{\bar{V}_{\mathrm{ig}}}{\bar{V}_{\alpha}}\right) + T\Delta^{\alpha}_{\mathrm{ig}}\bar{S}^{\mathrm{o}}_i(p-x) + RT\ln\left(\frac{\bar{V}_{\mathrm{ig}}}{\bar{V}_{\alpha}}\right) - \gamma_{\alpha}RT^2 + RT$$

$$\Delta\bar{H}^*_{i,\alpha} = \Delta^{\alpha}_{\mathrm{ig}}\bar{H}^{\mathrm{o}}_i(p-x) - \gamma_{\alpha}RT^2 + RT \qquad \text{(B11)}$$

where the relation $\Delta^{\alpha}_{\mathrm{ig}}\bar{H}^{\mathrm{o}}_i(p-x) = \Delta^{\alpha}_{\mathrm{ig}}\bar{G}^{\mathrm{o}}_i(p-x) + T\Delta^{\alpha}_{\mathrm{ig}}\bar{S}^{\mathrm{o}}_i(p-x)$ is used. The conversion factor for water at 25 °C is:

$$\begin{aligned} -\gamma_{\mathrm{w}}RT^2 + RT &= RT(-\gamma_{\mathrm{w}}T + 1) \\ &= 1.987\ \mathrm{cal\ K^{-1}\ mol^{-1}} \times 298.15\ \mathrm{K} \times (-3.657 \times 10^{-3}\ \mathrm{K^{-1}} \times 298.15\ \mathrm{K} + 1) \\ &= -0.05352\ \mathrm{kcal\ mol^{-1}} \end{aligned}$$

$$\Delta\bar{H}^*_{i,\alpha} = \Delta^{\mathrm{w}}_{\mathrm{ig}}\bar{H}^{\mathrm{o}}_i(p-x) - 0.05352\ \mathrm{kcal\ mol^{-1}} \qquad \text{(B12)}$$

This difference of -0.05352 kcal mol^{-1} is relatively small and, combined with the fact that $\Delta^{\alpha}_{\mathrm{ig}}\bar{H}^{\mathrm{o}}_i(p-x) = \Delta^{\alpha}_{\mathrm{ig}}\bar{H}^{\mathrm{o}}_i(c-c) = \Delta^{\alpha}_{\mathrm{ig}}\bar{H}^{\mathrm{o}}_i(p-b)$, the final conclusion is that the use of different concentration scales in the study of solvation enthalpies should not lead to significant differences, as concluded by Ben-Naim [3]. As far as transferences between different solvents α and β are concerned, the relation between $\Delta^{\beta}_{\alpha}\bar{H}^{\mathrm{o}}_i(p-x)$ and $\Delta^{\beta}_{\alpha}H^*_i$ is proportional to the difference between the thermal expansion coefficients of both phases, as shown in Eq. B9 for solvation entropies:

$$\Delta\bar{H}^*_{i,\beta} - \Delta\bar{H}^*_{i,\alpha} = \Delta^{\beta}_{\alpha}\bar{G}^{\mathrm{o}}_i(x-x) + RT\ln(\bar{V}_{\alpha}/\bar{V}_{\beta}) + T\Delta^{\beta}_{\alpha}\bar{S}^{\mathrm{o}}_i(x-x) + RT\ln(\bar{V}_{\alpha}/\bar{V}_{\beta}) - (\gamma_{\beta} - \gamma_{\alpha})RT^2$$

$$\Delta^{\beta}_{\alpha}\bar{H}^*_i = \Delta^{\beta}_{\alpha}\bar{G}^{\mathrm{o}}_i(x-x) + T\Delta^{\beta}_{\alpha}\bar{S}^{\mathrm{o}}_i(x-x) - (\gamma_{\beta} - \gamma_{\alpha})RT^2$$

$$\Delta^{\beta}_{\alpha}\bar{H}^*_i = \Delta^{\beta}_{\alpha}\bar{H}^{\mathrm{o}}_i(x-x) - (\gamma_{\beta} - \gamma_{\alpha})RT^2 \qquad \text{(B13)}$$

By considering the water/hexadecane mixture as an example:

$$\begin{aligned} (\gamma_{\mathrm{h}} - \gamma_{\mathrm{w}})RT &= 1.987\ \mathrm{cal\ K^{-1}\ mol^{-1}} \times (0.891 - 3.657) \times 10^{-3}\ \mathrm{K^{-1}} \times (298.15\ \mathrm{K})^2 \\ &= -0.489\ \mathrm{kcal\ mol^{-1}} \end{aligned}$$

$$\Delta^{\mathrm{h}}_{\mathrm{w}}\bar{H}^*_i = \Delta^{\mathrm{h}}_{\mathrm{w}}\bar{H}^{\mathrm{o}}_i(x-x) + 0.489\ \mathrm{kcal\ mol^{-1}} \qquad \text{(B14)}$$

The value 0.489 kcal mol^{-1} is quite small, usually below chemical precision, which implies that the experimental confrontation between solvation enthalpies from different standard states (by comparing process-x and process-c, for instance) through the partition of a solute between two immiscible liquids is considerably difficult, for the analytical method used should be very sensitive.

5. References

[1] Ben-Naim, A. Standard thermodynamics of transfer. Uses and misuses. *J. Phys. Chem.* **1978**, 82, 792–803.

[2] Moeser, B.; Horinek, D. The role of the concentration scale in the definition of transfer free energies. *Biophys. Chem.* **2015**, 196, 68–76.

[3] Ben-Naim, A. *Molecular theory of solutions*; Oxford University Press: Oxford, UK, 2006.

[4] Cohen, E.R.; Cvitas, T.; Frey, J.G.; Holmstrom, B.; Kuchitsu, K.; Marquardt, R.; Mills, I.; Pavese, F.; Quack, M.; Stohner, J.; Strauss, H.; Takami, M.; Thor, A.J. *Quantities, Units, and Symbols in Physical Chemistry*, *IUPAC Green Book*, 3rd ed.; RSC Publishing: London, UK, 2007.

[5] Abraham, M. H.; Whiting, G. S.; Fuchs, R.; Chambers, E. J. Thermodynamics of solute transfer from water to hexadecane. *J. Chem. Soc. Perkin Trans.* **1990**, 2, 291–300.
